# Work related determinants of breastfeeding discontinuation among employed mothers in Malaysia

**DOI:** 10.1186/1746-4358-6-4

**Published:** 2011-02-22

**Authors:** Rahmah Mohd Amin, Zakiah Mohd Said, Rosnah Sutan, Shamsul Azhar Shah, Azlan Darus, Khadijah Shamsuddin

**Affiliations:** 1 Department of Community Medicine, UKM Medical Centre, JalanYaakob Latif, 56000 Cheras, Kuala Lumpur, Malaysia; 2 Department of Social and Preventive Medicine, Universiti of Malaya, Malaysia

## Abstract

**Background:**

This cross-sectional study assesses factors that contribute to discontinuing breastfeeding among employed mothers in Malaysia.

**Methods:**

A structured questionnaire was used in conducting this study involving all government health clinics in Petaling district between July and September 2006. Respondents were Malaysian women with children between the ages of six to twelve months who were formally employed. Factors studied were selected socio-demographic and work-related characteristics.

**Results:**

From a total of 290 respondents, 51% discontinued breastfeeding. The majority (54%) of mothers who discontinued breastfeeding had breastfed their babies for less than three months. Compared to Malay mothers, the risk of breastfeeding discontinuation were higher among Chinese (AOR 3.7, 95% CI: 1.7, 7.8) and Indian mothers (AOR 7.3, 95% CI 1.9, 27.4). Not having adequate breastfeeding facilities at the workplace was also a risk factor for breastfeeding discontinuation (AOR 1.8, 95% CI: 1.05, 3.1).

**Conclusion:**

It is important that workplaces provide adequate breastfeeding facilities such as a room in which to express breast milk and a refrigerator, and allow mothers flexible time to express breast milk.

## Background

Increasing women's participation in the labour force is frequently blamed for the low rate of breastfeeding. Statistics from the Malaysian population and housing census showed an increasing trend in women's labour force participation from 30.8% (1957) to 47.7% (2003) [1]. The prevalence of *ever breastfed *declined from 92% (1950) to 78% (1974) but rose to 85% and 94.7% respectively in 1988 and 2006 [2,3]. The rise was explained by the introduction of the Malaysian Code of Ethics for Infant Formula Products in 1979. This code of ethics is to ensure the provision of safe and adequate nutrition among infants and an adequate standard and proper use of infant formula products. In addition, legal provisions were also included in the Food Regulations (1985) to promote breastfeeding [4]. Malaysian women employed in the public sector are given two months maternity leave while those working in the private sector are given a slightly longer leave of three months.

The Second National Health and Morbidity Survey (NHMS II) conducted in 1996 showed the prevalence of employed women who had ever breastfed was 91.4%. However, only 25.4% of employed women practiced exclusive breastfeeding compared to 31.3% among non-working women. The mean duration of breastfeeding were 26 weeks among working women compared to 30 weeks among those who were not working [2]. Breastfeeding may be less convenient for working women. Many factors determine the success of breastfeeding: a supportive workplace and working environment are essential.

In 1993, the Ministry of Health Malaysia adopted the WHO/UNICEF Baby Friendly Hospital Initiative. This initiative aimed to increase breastfeeding among all women in Malaysia in line with the WHO recommendation of at least six months of exclusive breastfeeding. Thus this study was conducted to assess the factors for discontinuation of breastfeeding among employed mothers. We examined the association between socio-demographic and workplace-related factors with discontinuation of breastfeeding among employed mothers.

## Methods

A cross-sectional study was conducted in Petaling district in Selangor. Selangor is a Malaysian state with one of the lowest breastfeeding rates [2]. It has the highest population density and has been recognized as one of the most developed states in Malaysia. Petaling district is one of the most urbanized and developed districts in the state of Selangor [1]. These factors were taken into consideration when selecting the study site.

All four governmental health clinics in Petaling were included in this study. All working mothers with children between the ages of three and twelve months who attended health services from these clinics were eligible for the study. Working mothers were defined as mothers who reported working for wages and had returned to work at the time of the interview. We recruited mothers with babies aged three months or more because maternity leave in Malaysia is up to three months and by then all working mothers would have returned to work. Mothers of children up to 12 months were also included because of limited time allocated for data collection. Mothers with twins or infants with congenital anomalies or those who were unable to breastfeed due to illness were excluded.

The structured self-administered questionnaire was developed in Malay (national) language and were pre-tested on a similar population. The estimated sample size calculated using Epi Info Version 6 based on prevalence of 79% of discontinued breastfeeding by six months was 255 [5]. We recruited equal number of mothers with Malaysian citizenship from all four clinics and at the end of the data collection period, 290 of the 300 (96.7%) mothers approached agreed to participate in the study.

In the analysis, we examined breastfeeding status at the time of data collection regardless of duration or exclusive or non-exclusive breastfeeding. For those who were no longer breastfeeding, we asked when they discontinued breastfeeding. In addition to mothers' ethnicity, education level and parity, the work-related factors explored were types of job (professional/managerial or non-professional/support staff/manual workers); types of employer (government, private or self-employed); work status reported as either full or part-time; work arrangement (fixed workplace or working mobile and working out-station). With regards to workplace facilities and policy, we asked questions on maternity, paternity and any other special breastfeeding leaves as well as arrangement for lighter job allocation or flexible working time given by the employer. For work-place breastfeeding facilities we asked questions on the availability of a nursery, whether mothers were given flexible time off work to express breast milk, availability of breast pump(s) and a room for expressing breast milk and refrigerator to store the milk.

We defined adequacy of breastfeeding facility at the workplace as having a place allocated for expressing breast milk and provision of flexible time to express breast milk other than the usual rest time. Descriptive, bivariate and multivariate analyses were carried out using SPSS version 12 to determine the socio-demographic and work related factors associated with breastfeeding. In the multivariate model, the predictors of discontinued breastfeeding were divided into socio-demographic and work related characteristics and workplace facilities and policies were included in the stepwise forward logistic regression analysis.

Approval from UKM Research and Ethics committee was obtained (FF-144-2006). All participants provided written consent. Data collection took place between July and September 2006.

## Results

Of the total 290 participants, 71% were aged between 25 and 35 years (mean age: 28.9 ± 4.3 years). The majority were Malays (74%) followed by Chinese (17%) and Indians (7%). With regards education, 56% had up to secondary education and 44% had diploma or higher level of education. One hundred and forty seven (51%) had only one child and 46% had 2 to 4 children. The majority (68%) were employed in the private sector and none were self-employed.

At the time of the study, 149 (51%) had discontinued breastfeeding. Among those who discontinued breastfeeding, 54% breastfed less than three months (80/149), 35% discontinued between three to six months (52/149), and only 12% discontinued after six months (17/149). Table 1 shows the relationship between specific socio-demographic and work related factors and breastfeeding status. More non-Malay mothers (76%) had discontinued breastfeeding compared to Malay mothers (43%) (p < 0.01).

**Table 1:** Association between breastfeeding practices with socio-demographic and work related characteristics

Characteristics		Breastfeeding status				OR
		Discontinued		Continued		(95% CI)
	Total	N	%	N	%	
**Ethnicity**						
Malays	215	92	42.8	123	57.2	0.24
Non-Malays	75	57	76.0	18	24.0	(0.13-0.43)
**Education level**						
Secondary school	162	87	53.7	75	46.3	1.24
Diploma and above	128	62	48.4	66	51.6	(0.78-1.96)
**Monthly income**						
<RM 2000	67	36	53.7	31	46.3	(ref)
RM2000-RM4999	171	89	52.0	82	48.0	0.94(0.53-1.65)
≥RM5000	52	24	46.2	28	53.8	0.74(0.36-1.53)
**No of Children**						
One	147	82	55.8	65	44.2	1.43
Two or more	143	67	46.9	76	53.1	(0.90-2.27)
**Categories of jobs**						
Professional	99	48	48.5	51	51.5	0.84
Non professional	191	101	52.9	90	47.1	(0.52-1.36)
**Type of employer**						
Government	94	38	40.4	56	59.6	0.52
Private	196	111	56.6	85	43.4	(0.32-0.86)
**Duration of work**						
< 6 years	175	91	52.0	84	48.0	1.07
≥ 6 years	115	58	50.4	57	49.6	(0.67-1.71)
**Working status**						
Full time	281	146	52.0	135	48.0	2.16
Part time	9	3	33.3	6	66.7	(0.53-8.82)
**Work arrangement**						
Fixed	263	137	52.1	126	47.9	1.36
Outstation	27	12	44.4	15	55.6	(0.61-3.02)

More mothers who worked in the private sector (57%) had discontinued breastfeeding at the time of the study than mothers who worked with the government (40%, p < 0.01).

Table 2 shows the association between breastfeeding and workplace support for breastfeeding. With regards to facilities, mothers who worked in workplaces that did not provide refrigerators were more likely to discontinue breastfeeding. Lack of flexible time to express breast milk was also associated with breastfeeding discontinuation (p < 0.01). In terms of related policies supportive of breastfeeding, 54% of mothers with longer maternity leave discontinued breastfeeding (p = 0.01). Other workplace and work-related policies like paternity leave, breastfeeding leave, arrangement for light duty after delivery and flexible working hours were not significantly associated with breastfeeding.

**Table 2:** Association between breastfeeding practice with workplace facilities and policies

Workplace facilities and policies		Breastfeeding status				OR
		Discontinued		Continued		(95% CI)
	Total	N	%	N	%	
**Flexible time to express breastmilk**						
Yes	132	55	41.7	77	58.3	0.49
No	158	94	59.5	64	40.5	(0.30-0.78)
**Room to express breast milk**						
Yes	130	60	46.2	70	53.8	0.68
No	160	89	55.6	71	44.4	(0.43-1.09)
**Refrigerator to keep breast milk**						
Yes	177	82	46.3	95	53.7	0.59
No	113	67	59.3	46	40.7	(0.37-0.96)
**Breast milk pump**						
Yes	9	2	22.2	7	77.8	0.29
No	267	133	52.8	134	47.7	(0.06-1.41)
**Nursery available**						
Yes	38	16	42.1	22	57.9	0.65
No	252	133	52.8	119	47.2	(0.33-1.30)
**Maternity leave**						
<2 months	21	5	23.8	16	76.2	0.27
≥2 months	269	144	53.5	125	46.5	(0.10-0.76)
**Paternity leave**						
Yes	215	106	49.3	109	50.7	0.72
No	75	43	57.3	32	42.7	(0.43-1.23)
**Breastfeeding leave**						
Yes	46	22	47.8	24	52.2	0.84
No	244	127	52.0	117	48.0	(0.45-1.59)
**Given lighter job**						
Yes	71	38	53.5	38	46.5	0.85
No	219	111	50.7	108	49.3	(0.49-1.45)
**Flexible working time**						
Yes	196	98	50.0	98	50.0	0.84
No	94	51	51.3	43	45.7	(0.52-1.38)

Multivariate analysis is shown in Table 3: the adjusted odds ratios for discontinuation of breastfeeding among employed mothers. Compared to Malay mothers, the risk of breastfeeding discontinuation were higher among Chinese (AOR 3.7, 95% CI: 1.7, 7.8) and Indian mothers (AOR 7.3, 95% CI 1.9, 27.4). Maternity leave of more than two months (AOR 5.2 95% CI: 1.7, 15.9) and not having adequate breastfeeding facilities at the workplace (AOR 1.8, 95% CI: 1.05, 3.1) were other risk factors for breastfeeding discontinuation.

**Table 3:** Multivariable analysis of discontinued breastfeeding among working women

Variables	Adjusted Odds Ratio	(95% CI)
**Ethnicity**		
[Malay]		
Chinese	3.7	(1.7-7.8)
Indian	7.3	(1.9-27.4)
Others	2.8	(0.5-16.7)
**Maternity leave**		
[< 2 months]		
≥2 months	5.2	(1.7-15.9)
**Types of employer**		
[Private]		
Government	0.8	(0.4-1.3)
**Breastfeeding facilities**		
[Adequate]*		
Inadequate	1.8	(1.1-3.1)

[ ] = reference

*availability of a room dedicated for breastfeeding and a refrigerator

## Discussion

In general, the socio-demographic pattern of women studied were similar to the Selangor state [1]. This study found that 51% of employed mothers were not breastfeeding their children at the time of data collection. This was almost similar to the prevalence of 50% found in the Second National Health and Morbidity Survey (NHMS) conducted by Ministry of Health Malaysia in 1996 [2]. The third NHMS conducted in 2006 found that the prevalence of complementary breastfeeding among infants below six months old was 48.2% [6]. Similarly, the finding from a study done in Singapore showed that even though 94.5% mothers attempted breastfeeding at one month, only 21.1% remained breastfeeding at six months [7]. A study carried out in Australia also showed that despite having high rate of breastfeeding initiation, this rate declined over time [8].

Based on the socio-demographic factors assessed in this study, ethnicity was found to be significantly associated with breastfeeding practice. Among those who discontinued breastfeeding, the majority were non-Malays. The Second NHMS also showed similar findings, where Malays were found to be more likely to breastfeed, followed by Indians and Chinese [2]. In Australia, the factors that were positively associated with breastfeeding at six months were having a strong desire to breastfeed, older maternal age, having been breastfed oneself as a baby and being born in an Asian country [8].

Multivariate analysis showed that working in the private sector was associated with a higher tendency to discontinue breastfeeding. In Thailand, Yimyam and colleagues found that urban women in the modern workplace faced many obstacles in their effort to maintain lactation [9]. In Malaysia, women working in the government sectors are more likely to have flexible time to express breast milk compared to those working in private sectors such as factory workers and other type of jobs with rigid and short resting periods. Furthermore, the government sector is more likely to adopt breastfeeding supportive policies. Malaysia has a breastfeeding policy which was developed in 1993 following the formation of the Breastfeeding Hospital Initiative Recognition committee. However how much such policy are being implemented by government or private sectors are still questionable because the obstacles of breastfeeding are not addressed by such policy.

Longer maternity leave resulting in better opportunity for breastfeeding was not supported by this study. This study found that those with longer maternity leave had higher risk of discontinuing breastfeeding compared to those with shorter leave. Despite receiving longer maternity leave they still discontinued breastfeeding in view of inadequate breastfeeding facilities at their workplaces. However there were only 21 women (7%) who had less than two months maternity leave; this small number limits the strength of these findings. Although there are work place based breastfeeding policy, the extent of implementation may differ by workplaces. Women who had longer maternity leave may work in places with less breastfeeding support and thus decided to stop breastfeeding earlier knowing that they cannot continue to breastfeed once they returned to work. This is often done to better establish their child nutrition and care arrangement after they returned to work.

Allowing some flexibility of working hours for mothers to express milk should not impose a strain on the job, as the time required to express their breast milk is minimal. Breastfed babies have been found to be less prone to illnesses and their mothers are less likely to miss work to take care of their sick child. Supportive environment and policies as well as providing facilities at the workplace have been promoted by the World Health Organization [10,11]. This study supported these elements since women working in workplaces providing less facilities were more likely to discontinue breastfeeding.

Availability of a refrigerator at the workplace was shown to be an important factor for maintaining breastfeeding among employed mothers in this study. Conservative estimates suggest that breast milk can be stored at room temperature for eight hours, refrigerated for up to eight days, and frozen for many months [12]. However, women will feel more confident if refrigerator is available for them to store the milk. Most women can express breast milk in less than an hour in two sessions when workplaces are supportive of breastfeeding [11].

One of the limitations in this study was that we did not collect the age of the infants at the time the mothers completed the survey; this could have helped explain the reasons for breastfeeding discontinuation. Information on the age of the infants would allow us to compare the results of this study with other studies.

## Conclusion

It is important that workplaces provide adequate breastfeeding facilities such as a room in which to express breast milk and a refrigerator, and allow mothers flexible time to express breast milk.

## Competing interests

The authors declare that they have no competing interests.

## Authors' contributions

RMA is the principal investigator. She and ZMS were involved in the initial preparation of the study design, analysis and writing-up. ZMS and AD were involved in the data collection as well as data entry. Data interpretation, analysis and writing were supported by SAS, KS and RS. All authors read and approved the final manuscript.
